# Exposure of Wheat Plants to Cerium Oxide Nanoparticles for Two Generations Affects the Third Generation’s Responses to Perfluorooctanesulfonic Acid

**DOI:** 10.1021/acsomega.5c06292

**Published:** 2025-10-01

**Authors:** Preston Clubb, Riley Pope-Buss, Maximo Reyes, Jessica Linson, Elim Horn, Jose Peralta-Videa, Illya Aidee Medina-Velo, Cyren M. Rico

**Affiliations:** † Chemistry and Biochemistry Department, 7471 Missouri State University, 901 S National Ave, Springfield, Missouri 65897, United States; ‡ 259111 Willard High School, 515 E Jackson St, Willard, Missouri 65781, United States; § Chemistry and Biochemistry Department, University of Texas at El Paso, 500 W University Ave, El Paso, Texas 79968, United States; ∥ Chemistry, Mathematics, and Physics Department, Houston Christian University, 7502 Fondren Rd, Houston, Texas 77074, United States

## Abstract

The effects of parental stress on the performance of the next generation plants exposed to another contaminant were investigated. Wheat plants were exposed to cerium oxide nanoparticles (CeO_2_–NPs) in the first and second generations and to perfluorooctanesulfonic acid (PFOS) in the third generation. Phenotypic or metabolic responses were assessed at 21 day (short-term exposure) or 90 day (long-term exposure) exposure periods. Biomass production, chlorophyll content, enzyme activity, and membrane damage were measured during short-term exposure, while elemental and PFOS concentrations and grain metabolites were analyzed during long-term exposure. Results showed that continued exposures to CeO_2_–NPs and PFOS improved chlorophyll content but reduced concentrations of important macro- and microelements in the grains of daughter plants. PFOS was accumulated in wheat grains, while metabolomic analysis revealed that the grain metabolite composition was significantly altered. Continued exposure to CeO_2_–NPs followed by exposure to PFOS decreased the abundances of most metabolites (22 out of 34). Consistent and repeated previous exposures to CeO_2_–NPs also progressively reduced the concentrations of sucrose-6-phosphate, adenine, glutamic acid, and other organic acid metabolites. The findings suggest that the prior generation’s exposure could still influence succeeding progeny generations via invisible changes in metabolite and elemental composition of grains.

## Graphical abstract

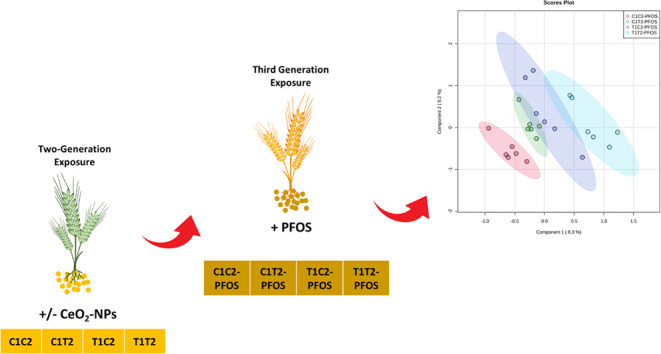

## Introduction

The use and disposal of industrially manufactured products have led to the release of numerous contaminants into the environment. While the list of environmental contaminants is extensive, cerium oxide nanoparticles (CeO_2_–NPs) and perfluorooctanesulfonic acid (PFOS) are of particular interest due to their stability in the environment, which directly impacts soil quality and plant growth.^1–4^ The effects of PFOS and CeO_2_–NPs on plants are diverse and depend on particle charge, concentration, plant species, growth stage, growth media, and environmental conditions. Several studies have measured their toxic effects in various plant species and growth media by measuring physiological, biochemical, molecular, and agronomic changes.^5–9^ Furthermore, it has also been widely reported that food crops and weeds (or spontaneous plants) can remove CeO_2_–NPs and PFOS from soil and water and store them in plant tissues.^3,9–15^

Generational exposures of plants to either NPs, PFOS, or other environmental contaminants have also been increasingly investigated.^4,16–24^ Interestingly, studies have revealed that multiple and successive exposures to contaminants can induce stress memory and elicit subtle phenological or phenotypical alterations instead of acute toxic effects, which in the end can modify the physiological traits and metabolome profile of the succeeding generation. Reports have shown that the maternal effects of different CeO_2_–NPs treatments have persisted to at least the second and third generations in seeds.^23,25^ The variability is present in different traits of F2 (i.e., second generation) populations from phenotypic variability and its components when exposed to TiO_2_–NPs.^24^ Regarding PFOS, accumulation decreased in second-generation soybean compared to the first-generation plants^16^ while the second-generation wheat grains had reduced abundances of select metabolites (e.g., sucrose, linolenic acid, ferulic acid) even when second-generation plants have been removed from PFOS exposure.^18^

Due to the imminent release of contaminants to the environment, agricultural soils are likely to be cocontaminated by legacy and/or emerging contaminants. Recent reports have explored the coexposure of two or more contaminants, including NPs with persistent pollutants such as fluorinated compounds.^26^ One study exposed radish (*Raphanus sativus*) to PFOA (4 mg/kg) and CuO NPs (200 and 400 mg/kg) for 30 days. They found that CuO NPs increased the transfer rate of PFOA from root to shoot, while PFOA reduced the toxic effects of CuO NPs in photosynthesis. ZnO NPs (100 mg/L) did not affect the uptake of PFOA in lettuce roots and shoots but reduced the uptake of GenX in lettuce roots.^27^ Similarly, Xu et al.^28^ reported that exposure to TiO_2_ NPs at low concentrations (0.05–5 mg/L) had minimal impacts on the uptake of PFOA/PFOS by hydroponically grown pumpkin seedlings. A similar study found that CeO_2_–NPs (100 mg/L) significantly enhanced pyrene uptake in soybean tissues (18.4–34.8%) after 15 days of exposure in hydroponics.^29^

Plant exposures to contaminants in successive generations have not yet been widely explored in the literature. In this study, the effects of multigeneration and successive exposures of wheat to CeO_2_–NPs (i.e., exposure in the first and second generations) and PFOS (i.e., exposure in the third generation) were investigated. Wheat was chosen as a model plant because it is an important food crop (the second most produced cereal) and a dietary staple for most of the world’s population. The goal was to assess whether parental exposures to CeO_2_–NPs impact the plant performance (i.e., physiology, yield, nutrient imbalances, and metabolomic processes) and PFOS accumulation in daughter wheat plants exposed to PFOS. Wheat grains harvested from CeO_2_–NPs exposure (500 mg/kg exposure treatment) in the first and second generations were exposed to PFOS in the third generation. The impact on plant health was assessed through several parameters, including biomass yield, enzyme activity, elemental accumulation, grain metabolites, and PFOS concentrations. The working hypothesis was that the continuous exposure of wheat to CeO_2_–NPs impairs the plant’s response to other contaminants such as PFOS.

## Materials and Methods

### Experimental Design and Treatment Application

Seedlings that had been exposed to CeO_2_–NPs for two generations from Rico et al.^4^ were exposed to PFOS (CAS # 1763-23-1, Sigma-Aldrich, St. Louis, MO) in the third generation under both short-term (21 days) and long-term (90 days) exposure conditions. Plants were exposed to four treatments: C1C2-PFOS, C1T2-PFOS, T1C2-PFOS, and T1T2-PFOS, where C = untreated control, T = treated with 500 mg CeO_2_–NPs per kg soil, 1 and 2 = first and second generations, respectively, and PFOS = treated with 50 mg/kg PFOS in the third generation (Figure 1). Each treatment had six pots (replicates) containing two seedlings. The soil was a mixture of 2.2:1 (v:v) ProMix HP Mycorrhizae and sand. Each pot had 0.8 and 2.4 kg of mixed soil for the short- and long-term studies, respectively. PFOS was added to the soil 3 days before transplanting the wheat germinates. Plants were grown in a greenhouse under controlled conditions of 14 h photoperiod, 25 °C/20 °C Day/Night temperature, 65–75% relative humidity, and a daylight integral (DLI) of 30 mol (400 PAR).

**Figure 1:**
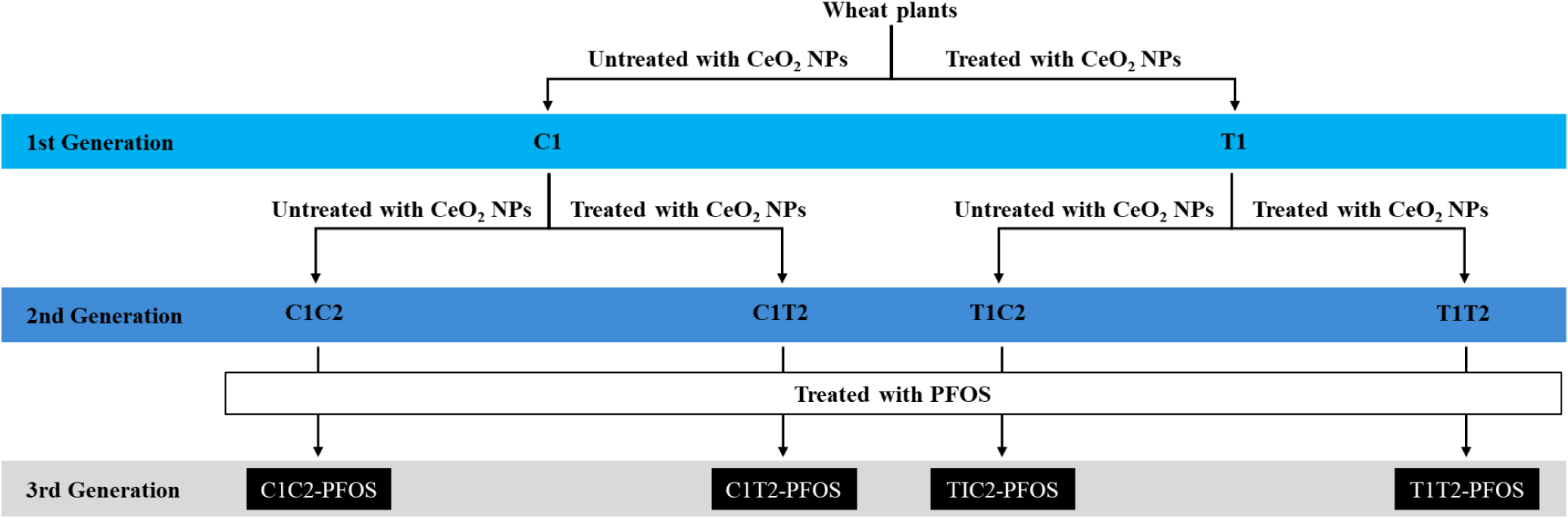
Experimental treatments were C1C2-PFOS, C1T2-PFOS, T1C2-PFOS, and T1T2-PFOS, where C = untreated, T = treated with 500 mg of CeO_2_–NPs per kg of soil, and 1 and 2 = 1st and 2nd generations, respectively; PFOS = treated with 50 mg/kg perfluorooctanesulfonic acid (PFOS) in the 3rd generation.

### Plant Cultivation

All of the glassware and double deionized (DDI) water used for the experiment were autoclaved. Wheat seeds utilized were harvested from the second-generation experiment by Rico et al.^4^ Seeds were disinfected in a Petri dish by immersion in 5% solution of commercial bleach for 15 min, followed by three rinsing cycles with 5 mL of DDI. Then, seeds were immersed in liquid commercial copper fungicide (Bonide) for 15 min followed by rinsing with DDI. To promote seed germination, around 2 mL of DDI was added to the seeds in the Petri dish and then transferred to an incubator at 25 °C for 3 days. After 3 days, 5 mL of Yoshida Nutrient Solution was added to the Petri dish, and the Petri dish was transferred to a growth chamber set at 25 °C and 40% humidity for 4 days to germinate. Plant germinants were then ready to be transplanted into the PFOS-containing soil.

DDI water (100 mL) was used to wet the previously prepared PFOS-containing soil before transferring the pots to the greenhouse. Subsequently, two seedlings were transplanted into each pot. The plants were fertilized with 50 mL of Yoshida Nutrient Solution once a week for the short-exposure study (21 days) and the long-term study (90 days). The pots were set in a randomized block design, and their positions were rotated regularly.

### Biochemical Assays and Agronomic Parameters Measurement

Plant height and biomass in both exposure scenarios were recorded. Grain yield was also recorded from the long-term exposure plants. Fresh leaves for biochemical assays (e.g., chlorophyll, enzyme, and stress assays) were collected, frozen in liquid nitrogen, and stored at −80 °C until they were analyzed. The grains were also collected and kept in a −80 °C freezer for metabolomics and PFOS analysis. The remaining samples were oven-dried (70 °C), ground using a ball mill grinder (Retsch MM 200, Newton, PA), and stored at room temperature for elemental analysis.

### Chlorophyll Content Determination

Chlorophyll content was determined following a previous method.^30^ Immediately following the harvest of the short-exposure wheat, 0.1 g of fresh leaves from each treatment were soaked in 5 mL of 70% ethanol. The tubes were placed on a rocker (Fisher Scientific) for 24 h in the dark. The absorbances at 665 and 649 nm were measured using a UV–visible spectrophotometer (Cary 60, Agilent, Santa Clara, CA).

### Catalase Activity and Ascorbate Peroxidase Activity

The determination of catalase activity followed the method described by Ofoegbu et al.^31^ A fresh sample (0.5 g) of wheat shoots from the short exposure (21 days) was homogenized and centrifuged, and the extract was analyzed for catalase (CAT) and ascorbate peroxidase (APOX) enzymatic activities using a UV–visible spectrophotometer (Cary 60, Agilent, Santa Clara, CA).

### Lipid Peroxidation

Lipid peroxidation was determined following the procedure reported by Gay and Gebicki.^32^ A sample (0.5 g) of wheat roots and shoots from the short exposure (21 days) was homogenized in 2 mL of 0.1% trichloroacetic acid (TCA) and centrifuged for 20 min at 5000 rpm. The supernatant was collected, followed by the addition of 100 μL of butylated hydroxytoluene (BHT) and 1 mL of thiobarbituric acid (TBA). The mixture was heated at 95 °C for 30 min followed by 15 min centrifugation at 5000 rpm. The supernatant was collected, and absorbances at 532 and 600 nm were recorded using a UV–visible spectrophotometer (Cary 60, Agilent, Santa Clara, CA).

### Element Determination

Each dry ground sample weighing 0.25 g underwent acid digestion in a microwave digester (CEM Mars 6^TM^, Matthews, NC) using 5 mL of 70% plasma pure nitric acid (HNO_3_, SCP Sciences, Champlain, NY). The digestates were transferred to a centrifuge tube and then diluted to 50 mL using DDI, followed by a second dilution with DDI to achieve a 2% acid content before analysis. Measurement of Peach leaves (NIST 1547; Gaithersburg, MD, USA) was used as a standard reference material. A 7900 Agilent Technologies ICP-MS instrument was used for the elemental analysis.

### Metabolomics

Fiehn Lab at the West Coast Metabolomics Center, University of California, Davis performed the metabolomics analysis using gas chromatography-quadrupole time-of-flight mass spectrometry. Metabolomics determination followed the method reported by Ofoegbu et al.^31^ following the sample treatment, methodology, and instrumentation described by Fiehn et al.^33^ Original data points with confidence levels below 10% were excluded from further analysis in MetaboAnalyst 5.0. Metabolites with variable importance in projection (VIP) values exceeding one were regarded as significant.

### PFOS Analysis

GEL Laboratories, LLC (Charleston, SC) performed the extraction and analysis of PFOS in dry, ground wheat root, shoot, and grain samples using liquid chromatography with tandem mass spectrometry (LC-MS-MS) following a method modified from EPA 573.1.

### Data Analysis

The data were examined using Tukey’s multiple range model in the SAS statistical software (SAS Institute, Cary, NC) after a one-way analysis of variance (ANOVA) test at *p* < 0.05. Samples were analyzed in six replicates, and the mean and standard errors were calculated. MetaboAnalyst software was used for multidimensional metabolomics analysis, which included a partial least-squares discriminant analysis (PLS-DA) plot.

## Results and Discussion

### Plant Productivity

Wheat growth and yield performance were evaluated under short-term and full-lifecycle exposures to PFOS (Table 1). The biomass yields of the roots, shoots, and grains did not vary between treatments, indicating that continuous exposure to CeO_2_–NPs and PFOS did not affect plant biomass production (Table 1). Our previous report on generational studies also showed no differences in plant productivity during full life cycle exposure to CeO_2_–NPs.^4^ Medina-Velo et al.^22^ also found that the yield of daughter plants of common bean (*Phaseolus vulgaris*) grown in soil devoid of ZnO-NPs (0–0 mg/kg ZnO-NPs) was unaffected by parental exposure to ZnO-NPs (500–0 mg/kg ZnO-NPs). Conversely, in a comparable study, Shimalina et al.^19^ discovered consistent effects of reduced root growth in second- and third-generation *Plantago major* plants grown in soil free of ionizing radiation (i.e., first-generation plants were exposed to radiation), demonstrating a durable transgenerational influence on root growth.^31^ They also reported a decreasing trend in grain biomass in wheat exposed to PFOS compared to untreated control; such a trend was not observed in the current study, further confirming that continuous exposure to CeO_2_–NPs, i.e., parental exposures to CeO_2_–NPs, did not affect plant productivity. Current findings support the idea that longer exposure studies would provide an improved understanding of the impacts of pollutants on plant growth and productivity.

**Table 1:** Dry Biomass Production (g) of Wheat Generationally Exposed to Cerium Oxide Nanoparticles and Perfluorooctanesulfonic Acid for 90 Days^a^

	C1C2-PFOS	C1T2-PFOS	T1C2-PFOS	T1T2-PFOS
Short-term exposure				
**Shoot**	0.244 ± 0.03a	0.223 ± 0.02a	0.285 ± 0.03a	0.209 ± 0.01a
**Root**	0.483 ± 0.06a	0.427 ± 0.04a	0.532 ± 0.06a	0.439 ± 0.03a
Long-term exposure				
**Grain**	7.45 ± 0.30a	8.11 ± 0.30a	7.77 ± 0.20a	6.78 ± 0.70a
**Shoot**	5.86 ± 1.26a	5.59 ± 0.70a	5.69 ± 0.83a	5.83 ± 0.97a
**Root**	2.26 ± 0.81a	2.29 ± 0.52a	2.00 ± 0.51a	2.58 ± 0.54a

a Values are mean ± SE (*n* = 6). Same letters across treatment groups indicate no statistical difference (*p* < 0.05). Refer to Figure 1 for the explanation of the treatments.

### Biochemical Indices

Chlorophyll concentration, lipid peroxidation, and enzyme activity were determined as measures of stress under short-exposure regimen (Figures 2 and 3). Chlorophyll *a* concentration increased in T1C2-PFOS and T1T2-PFOS compared to C1C2-PFOS, but there was no change in the chlorophyll *b* concentration across treatments (Figure 2A). Notably, chlorophyll *a* concentration increased progressively by 23%, 36%, and 45%, respectively, in plants previously exposed to CeO_2_–NPs (i.e., C1T2-PFOS, T1C2-PFOS, and T1T2-PFOS) compared to the control (i.e., C1T2-PFOS). Lipid peroxidation (LPOX), a measure of oxidative stress, and catalase activity (CAT), a measure of antioxidant enzyme activity, were not affected across treatments (Figures 2B and 3A). Ascorbate peroxidase (APOX) activity exhibited an increasing trend in plants previously exposed to CeO_2_–NPs (C1T2-PFOS, T1C2-PFOS, and T1T2-PFOS) by 7%, 11%, and 37%, respectively, compared to the control (C1C2-PFOS) (Figure 3B). However, a significant increase was recorded in T1T2-PFOS vs C1C2-PFOS only (Figure 3B). These data showed that generational exposures to contaminants (i.e., CeO_2_–NPs and PFOS) could increase chlorophyll *a* and APOX activity at the juvenile stage of the plant. Since the biomasses were not affected (Table 1), this improved performance at the young growth stage of the plant was highly temporal and not sustained until the end of long-term plant growth.

**Figure 2:**
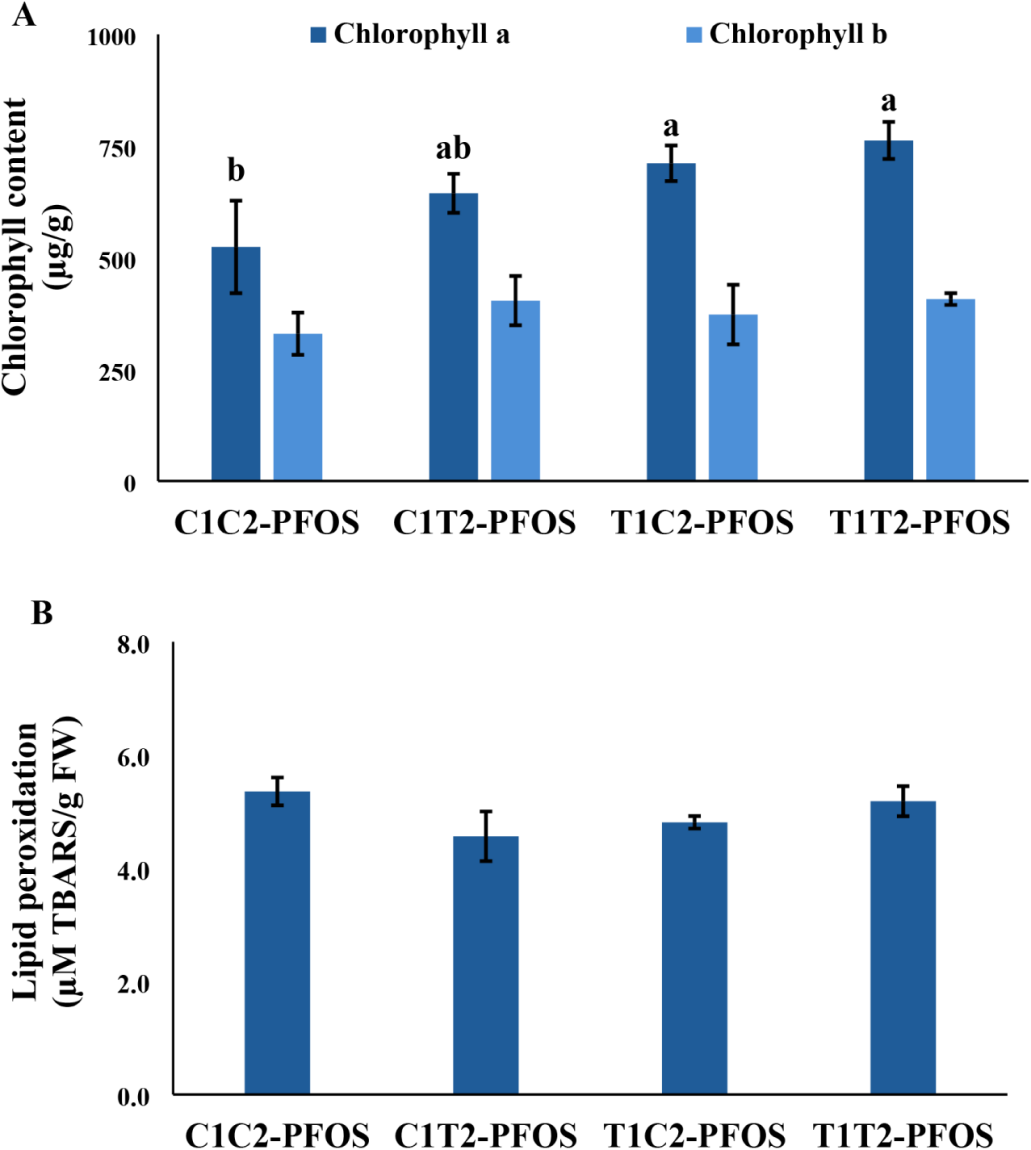
Stress levels measured as (A) chlorophyll content and (B) lipid peroxidation in wheat generationally exposed to CeO_2_–NPs and PFOS for 21 days. Values are mean ± SE (*n* = *6*). Different letters across treatments indicate significant difference (*p* < 0.05). Refer to Figure 1 for the explanation of the treatments.

**Figure 3:**
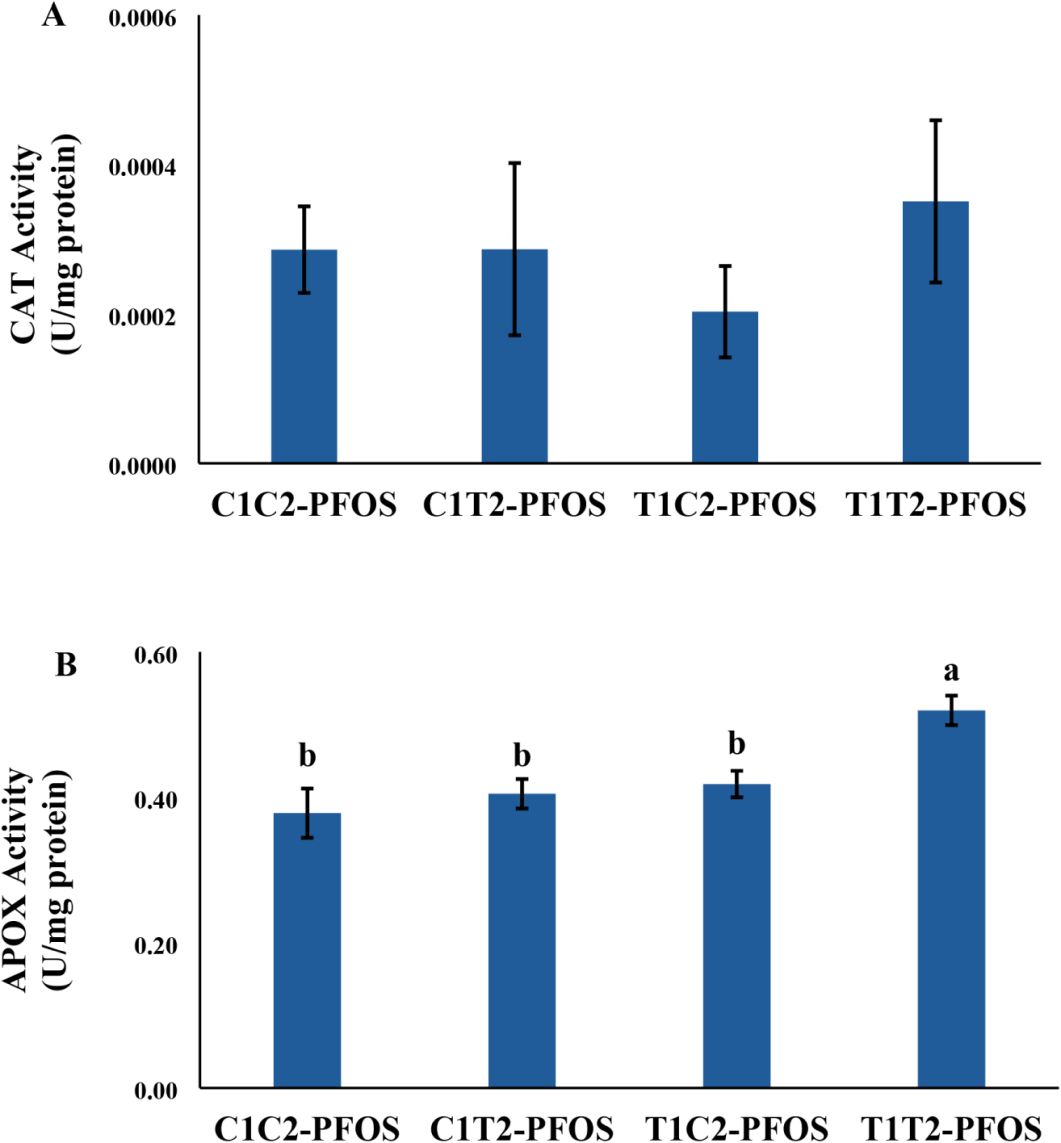
Enzyme activity of (A) catalase (CAT) and (B) ascorbate peroxidase (APOX) in wheat generationally exposed to CeO_2_–NPs and PFOS for 21 days. Values are mean ± SE (*n* = *6*). Different letters across treatments indicate significant difference (*p* < 0.05). Refer to Figure 1 for the explanation of the treatments.

Research published on the generational impact of nanoparticles (NPs) on plant chlorophyll concentration differs significantly from our observations. According to a study by Tan et al.,^21^ TiO_2_–NPs did not influence the amount of chlorophyll *a* in basil (*Ocimum basilicum*) after two generations. Medina-Velo et al.^22^ also found that, compared to control plants (0–0 mg/kg ZnO-NPs), the activity of the enzymes CAT, APOX, and superoxide dismutase (SOD) was unaffected in beans previously exposed to ZnO-NPs (500 mg/kg ZnO-NPs). *Plantago major* showed signs of oxidative stress through LPOX even after stress was removed in the second and third generations, but only in cases where parent plants had been subjected to extremely high levels of ionizing radiation.^19^ These researchers also reported that among the second- and third-generation stress-free plants, CAT and SOD enzyme activity did not maintain a consistent trend. Additionally, Wang et al.^20^ observed no differences between control and second-generation tomato seedlings cultivated in clean soil (10–0 mg/L CeO_2_–NPs) regarding the impact of prior generation exposure to CeO_2_–NPs on oxidative stress via H_2_O_2_ production. Moreover, Ma et al.^34^ found that at 1000 mg/L CeO_2_–NPs, third-generation *Brassica rapa* had higher CAT activity and MDA concentration (a measure of LPOX) than earlier generations. Physiological and biochemical indices at the short-term exposure or juvenile stage do not provide strong evidence of plant responses to continuous exposures of plants to contaminants. Assessing any potential effects that NPs may have on future generations at the juvenile stage may require more sophisticated techniques, such as epigenetics.

### Uptake of PFOS

PFOS concentration in grains, shoots, and roots did not change between treatments except for a significant 46% increase in PFOS concentration in C1T2-PFOS vs C1C2-PFOS (Figure 4). Notably, the trend for grain PFOS concentration seemed to show a “U-shaped” trend across treatments (Figure 4A). Previous studies also showed the accumulation of PFOS in plant roots, shoots, and grains^31^ Nevertheless, the process by which plants enable PFOS to enter their roots and travel via the xylem and phloem streams to eventually accumulate in their shoots and grains remains unclear. Many parameters specific to plants, such as physiology, root anatomy, chemical compositions, water or anion channels, and xylem-to-phloem transfer, have been proposed by researchers as factors influencing PFOS uptake.^11,35–38^ Environmental factors, such as ambient temperature, light intensity, humidity, and soil organic content, were also considered.^8,36,39^ A recent study by Qian et al.^40^ shows that short-chain hydrophobic compounds are rapidly translocated upward after absorption. Still, long-chain hydrophobic compounds are likely to be absorbed and kept on the root epidermis. When exposed to high concentrations, the bioaccumulation of per- and polyfluoroalkyl substances (PFAS) was shown to be higher in leaves than in stems, and the bioaccumulation decreased as the C–F chain length decreased.^41^ On the other hand, Liu et al.^42^ discovered that the slow-type anion channel pathways that interact with Ca^2+^-dependent protein kinases (Ca^2+^-CDPK-SLAC1) are primarily responsible for the transport of PFAS triggered by humic acid. While the mechanism of PFOS is slowly being understood, data from the current study suggest that prior exposure to CeO_2_–NPs has no dramatic impact on the translocation of PFOS from roots to shoots and grains.

**Figure 4:**
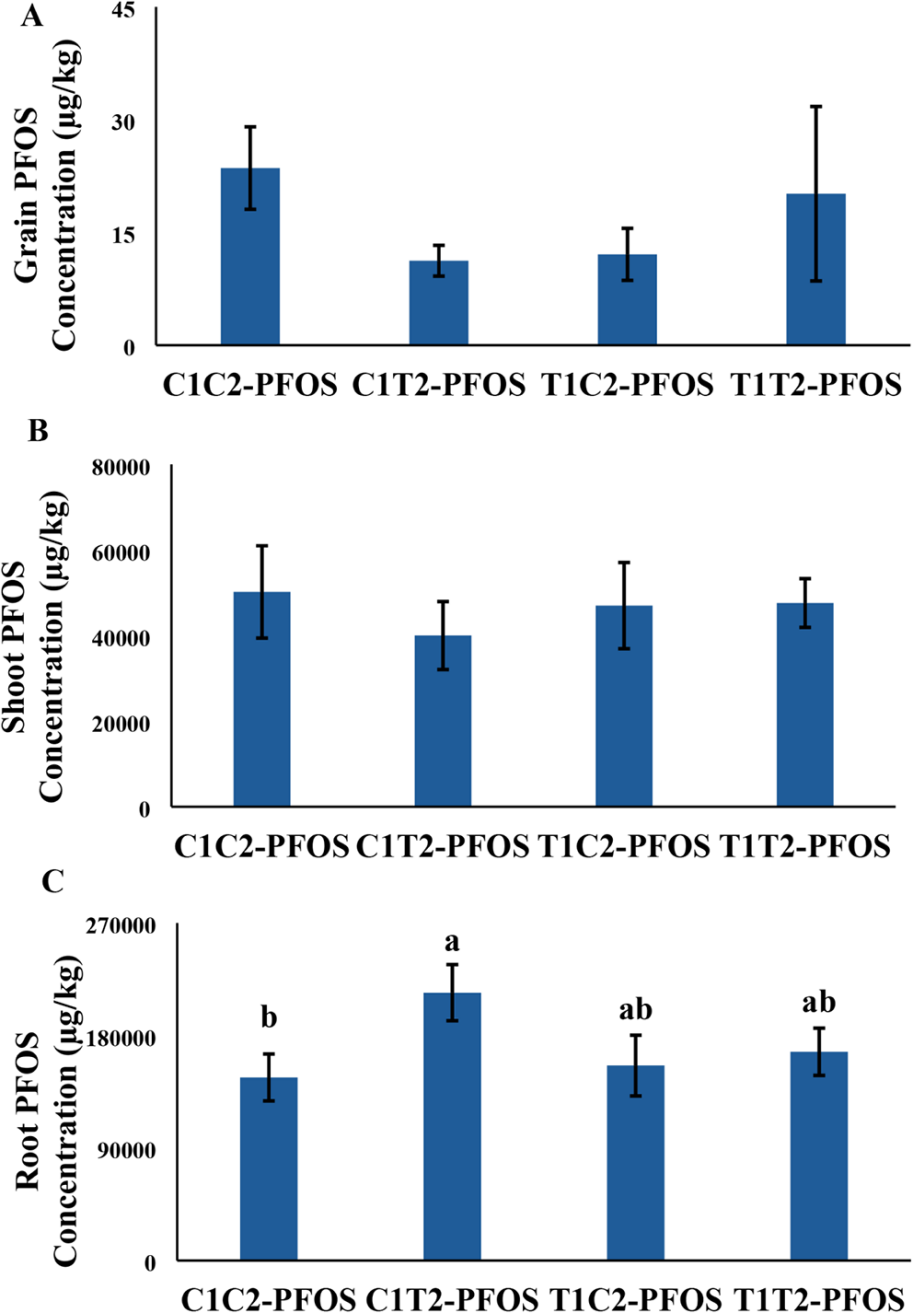
Perfluorooctanesulfonic acid (PFOS) concentration in (A) grains, (B) shoot, and (C) (root) of wheat generationally exposed to CeO_2_–NPs and PFOS for 90 days. Values are mean ± SE (*n* = *6*). Different letters across treatments indicate significant difference (*p* < 0.05). Refer to Figure 1 for the explanation of the treatments.

### Macro- and Microelements Uptake

The elemental uptake or accumulation in the roots and shoots is presented in the Supporting Information (Tables S1 and S2). In roots, Mg, Mn, Fe, and Co concentrations increased (by 24%, 69%, 36%, and 58%, respectively), while S and Cu concentrations decreased (by 16% and 79%, respectively) in T1T2-PFOS compared to C1C2-PFOS (Table S1). Interestingly, the concentrations of macroelements in the shoots did not change between treatments (Table S2). However, B, Si, and Mn concentrations increased (by 32%, 105%, and 50%, respectively), while Fe and Mo concentrations decreased (by 20% and 70%, respectively) in T1T2-PFOS compared to C1C2-PFOS (Table S2).

In grains, concentrations of all elements (except for Si and Co which did not change between treatments) exhibited a strong tendency to decrease when the plants were continuously exposed to CeO_2_–NPs and PFOS (Table 2). Some elemental concentrations tended to be similar to those of the control when the plants were previously exposed to T1T2-PFOS, exhibiting a “U-shaped” trend in elemental concentrations. For example, the concentrations of Mg, P, and K in C1T2-PFOS and T1C2-PFOS were lower by 23–32%, 18–28%, and 24–32%, respectively, compared to C1C2-PFOS (Table 2). However, their concentrations in T1T2-PFOS increased to be statistically similar to C1C2-PFOS. A similar “U-shaped” trend was also observed in PFOS accumulation in grains (Table 2 and Figure 4A). In general, the results indicated that the grains were more susceptible to alterations in elemental concentration, since changes in the elemental concentration in the shoots and roots were very minimal. This trend suggests that the biological processes governing the relocation of elements from shoots to grains were affected by continuous exposures to CeO_2_–NPs and PFOS. Our previous study also showed that the grain accumulation of elements was susceptible to parental exposure to CeO_2_–NPs.^25,43^ Previous studies on PFOS and plants have found that, except magnesium, most elements’ root-to-shoot translocation does not appear to be significantly threatened by PFOS^31,44,45^ while PFOS caused a decrease in the accumulation of Mg, P, and K in wheat grains.^31^

**Table 2:** Grain Elemental Concentration (mg/kg) of Wheat Generationally Exposed to Cerium Oxide Nanoparticles and Perfluorooctanesulfonic Acid for 90 Days^a^

Elements	C1C2-PFOS	C1T2-PFOS	T1C2-PFOS	T1T2-PFOS
Mg	1204 ± 81a	924 ± 78b	816 ± 42b	1017 ± 76ab
P	2817 ± 192a	2310 ± 143bc	2031 ± 135c	2519 ± 145ab
K	3737 ± 344a	2858 ± 224b	2535 ± 105b	3134 ± 281ab
Ca	389 ± 75a	189 ± 18ab	140 ± 19b	160 ± 20b
S	1228 ± 128a	745 ± 75ab	707 ± 40b	860 ± 89ab
B	1.07 ± 0.09a	0.94 ± 0.11a	0.28 ± 0.05b	0.35 ± 0.06b
Cu	2.9 ± 0.22a	2.4 ± 0.17ab	1.74 ± 0.14c	2.18 ± 0.2bc
Si	23.5 ± 4.7a	20.5 ± 6.3a	18.3 ± 2.3a	17.1 ± 4.1a
Mn	23.2 ± 2.8a	18.1 ± 1.4ab	14.2 ± 1.4b	18.6 ± 1.1ab
Zn	24.4 ± 2.2a	20.6 ± 2.4ab	14.9 ± 1.4b	17.3 ± 1.7b
Fe	40.1 ± 5.6a	28.3 ± 4.2b	6.6 ± 1.4c	18 ± 3.3bc
Co	0.03 ± 0a	0.03 ± 0a	0.03 ± 0a	0.03 ± 0a
Mo	0.20 ± 0.03a	0.12 ± 0.01b	0.12 ± 0.01b	0.13 ± 0.01b

a Values are mean ± SE (*n* = 6). Different letters across treatments indicate significant differences (*p* < 0.05). Refer to Figure 1 for the explanation of the treatments.

It is interesting to note that there were limited effects on elemental uptake in the roots and shoots compared to those in the grains. These findings indicate that exposure to the contaminant did not affect the entry of elements into the root and the root-to-shoot movement of these elements. However, the shoot-(particularly the flag leaf)-to-seed transport of the elements is much more sensitive to the precise programming and timing of various physiological processes during grain filling.^46,47^ Overall, the current data revealed that continuous exposure to CeO_2_–NPs and PFOS could negatively impact the elemental accumulations in grains.

### Grain Metabolomics

Grain metabolome is a measure not only of grain quality but also of the reserve mobilization from leaves (i.e., flag leaves) to the developing grains/seeds.^47^ Hence, grain metabolome could be an indicator of molecular and metabolic activities in plants occurring from early to later maturation.^48^ The metabolomics analysis identified 165 metabolites, and partial least-squares discriminant analysis (PLS-DA) revealed that previous generation exposures to CeO_2_–NPs modified the metabolite profile of PFOS-exposed wheat grains (Figure 5 and Tables 3 and 4). The ellipses representing previous exposure to CeO_2_–NPs (C1T2, T1C2, and T1T2) were well separated from the control group (C1C2) (Figure 5). The scores plot also indicates that T1T2 had a more significant impact on the metabolite profile of wheat grains compared to C1T2 or T1C2. The PLS-DA gave 34 metabolites with variable importance in projection (VIP) scores > 1 (Tables 3 and 4).Pathway analysis revealed that parental exposure to CeO_2_–NPs perturbed three metabolic pathways in grains of PFOS-exposed wheat plants: starch and sucrose metabolism, tyrosine and glutamine metabolism, and purine metabolism. The 34 differential metabolites were used to understand the metabolomic changes in wheat grains. Figure 6 provides a map of the pathway of the affected metabolites.

**Figure 5:**
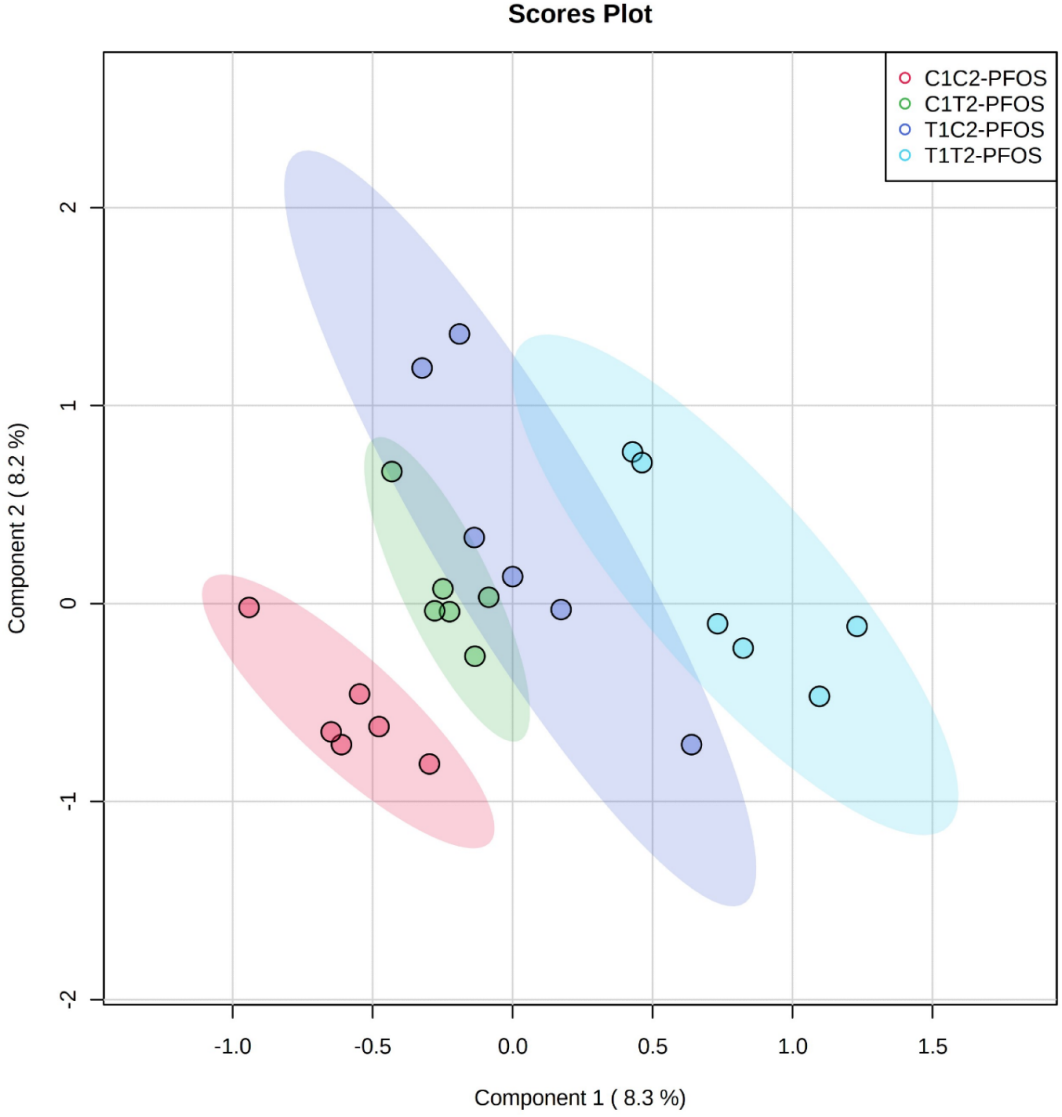
Partial least-squares-discriminant analysis (PLS-DA) of grains of wheat generationally exposed to CeO_2_–NPs. Analysis was performed using MetaboAnalyst 5.0. Refer to Figure 1 for the explanation of the treatments.

**Table 3:** Changes in Metabolite Abundances of Sugars and Organic Acids in Wheat Grain Generationally Exposed to Cerium Oxide Nanoparticles and Perfluorooctanesulfonic Acid for 90 Days^a^

Metabolite	C1C2-PFOS	C1T2-PFOS	T1C2-PFOS	T1T2-PFOS
Sugars				
1-kestose	2141650 ± 1006853	2633329 ± 439109↑	2525966 ± 801689↑	2680354 ± 172727↑
1,2,4-butanetriol	8042 ± 3584	6328 ± 886↓	6089 ± 999↓	5329 ± 768↓
5-deoxyribitol	11458 ± 3418	8452 ± 2248↓	7466 ± 2770↓	9286 ± 2985↓
Glucoheptulose	6750 ± 3273	5906 ± 2798↓	7740 ± 3324↑	8259 ± 1836↑
Leucrose	2942 ± 1296	1433 ± 368↓	1737 ± 534↓	1281 ± 431↓
Lyxose	19763 ± 14466	25536 ± 19999↑	13211 ± 2706↓	12754 ± 2309↓
Maltitol	2392 ± 649	2551 ± 377↑	2306 ± 432↓	2083 ± 805↓
Maltotriitol	97345 ± 50410	75863 ± 34654↓	76186 ± 37980↓	60705 ± 33918↓
Sucrose-6-phosphate	1668 ± 216	1601 ± 238↓	1441 ± 318↓	1348 ± 330↓
Organic Acids				
Glucaric acid	130860 ± 17979	127422 ± 30147↓	178764 ± 44816↑	121359 ± 37572↓
Gluconic acid	5238 ± 640	3684 ± 361↓	3345 ± 883↓	3213 ± 580↓
Itaconic acid	23457 ± 4050	16309 ± 7397↓	16218 ± 3127↓	17003 ± 4484↓
Lactobionic acid	9554 ± 7024	6182 ± 2906↓	10789 ± 4548↑	12123 ± 2264↑
Mannonic acid	1816 ± 792	1606 ± 244↓	1882 ± 740↑	1930 ± 255↑
Xylonic acid	10889 ± 7794	9144 ± 7100↓	7751 ± 5900↓	4715 ± 1855↓

a Values are means ± SE (*n* = 6). Up (↑) or down (↓) arrows indicate an increase or decrease in treated grains (C1T2-PFOS, T1C2-PFOS, and T1T2-PFOS) relative to the control (C1C2-PFOS) as measured by variable importance in projection (VIP > 1). Refer to Figure 1 for the explanation of the treatments.

**Table 4:** Changes in Metabolite Abundances of Amino Acids, Lipids, and Nucleic Acids in Wheat Grain Generationally Exposed to Cerium Oxide Nanoparticles and Perfluorooctanesulfonic Acid for 90 Days^a^

Metabolite	C1C2-PFOS	C1T2-PFOS	T1C2-PFOS	T1T2-PFOS
Amino acids				
2-oxoadipate acid	3693 ± 1668	2894 ± 788↓	2664 ± 669↓	2428 ± 1020↓
α-aminoadipic acid	9040 ± 3223	5684 ± 935↓	5235 ± 804↓	6852 ± 1977↓
Glutamic acid	1052498 ± 271782	930070 ± 87104↓	869665 ± 163775↓	853348 ± 274512↓
Glutamine	97763 ± 59784	63463 ± 39231↓	93728 ± 56829↓	108181 ± 135786↑
Methionine	6292 ± 1145	5938 ± 727↓	6180 ± 995↓	8700 ± 3871↑
N-Acetylserine	13391 ± 17812	6195 ± 4804↓	4100 ± 2070↓	5009 ± 2021↓
Tryptophan	742590 ± 435348	882244 ± 440004↑	1186415 ± 698582↑	1538935 ± 833337↑
Tyrosine	145700 ± 35938	140744 ± 26787↓	154852 ± 21423↑	189601 ± 30116↑
Lipids				
Campesterol	71704 ± 29005	56772 ± 9837↓	60815 ± 6362↓	53803 ± 21812↓
Docosanoic acid	1546590 ± 693230	1987005 ± 194707↑	1709691 ± 789449↑	643118 ± 790286↓
Glyceryl monooleate	176087 ± 153252	105470 ± 116780↓	128238 ± 78514↓	107972 ± 105266↓
Glyceryl monostearate	8558 ± 7220	2999 ± 2178↓	4604 ± 1257↓	6684 ± 1049↓
Glyceryl palmitate	8535 ± 4594	6250 ± 480↓	6124 ± 1165↓	5869 ± 1423↓
Hexanoic acid	3755 ± 2989	2241 ± 509↓	2564 ± 920↓	2199 ± 243↓
Icosanoic acid	19740 ± 8801	13453 ± 4883↓	16579 ± 1690↓	10140 ± 3702↓
Nonadecanoic acid	9815 ± 3992	7266 ± 1261↓	7290 ± 2051↓	6868 ± 733↓
Octacosanoic acid	15725 ± 6226	10807 ± 3584↓	15233 ± 4230↓	10906 ± 5454↓
Nucleic acids				
Adenine	7025 ± 979	5410 ± 627↓	5336 ± 619↓	5264 ± 745↓
Adenosine-5-monophosphate	2878 ± 678	2705 ± 876↓	2829 ± 913↓	9592 ± 1677↑

a Values are means ± SE (*n* = 6). Up (↑) or down (↓) arrows indicate an increase or decrease in treated grains (C1T2-PFOS, T1C2-PFOS, and T1T2-PFOS) relative to control (C1C2-PFOS) Table 2. Refer to Figure 1 for the explanation of the treatments.

**Figure 6:**
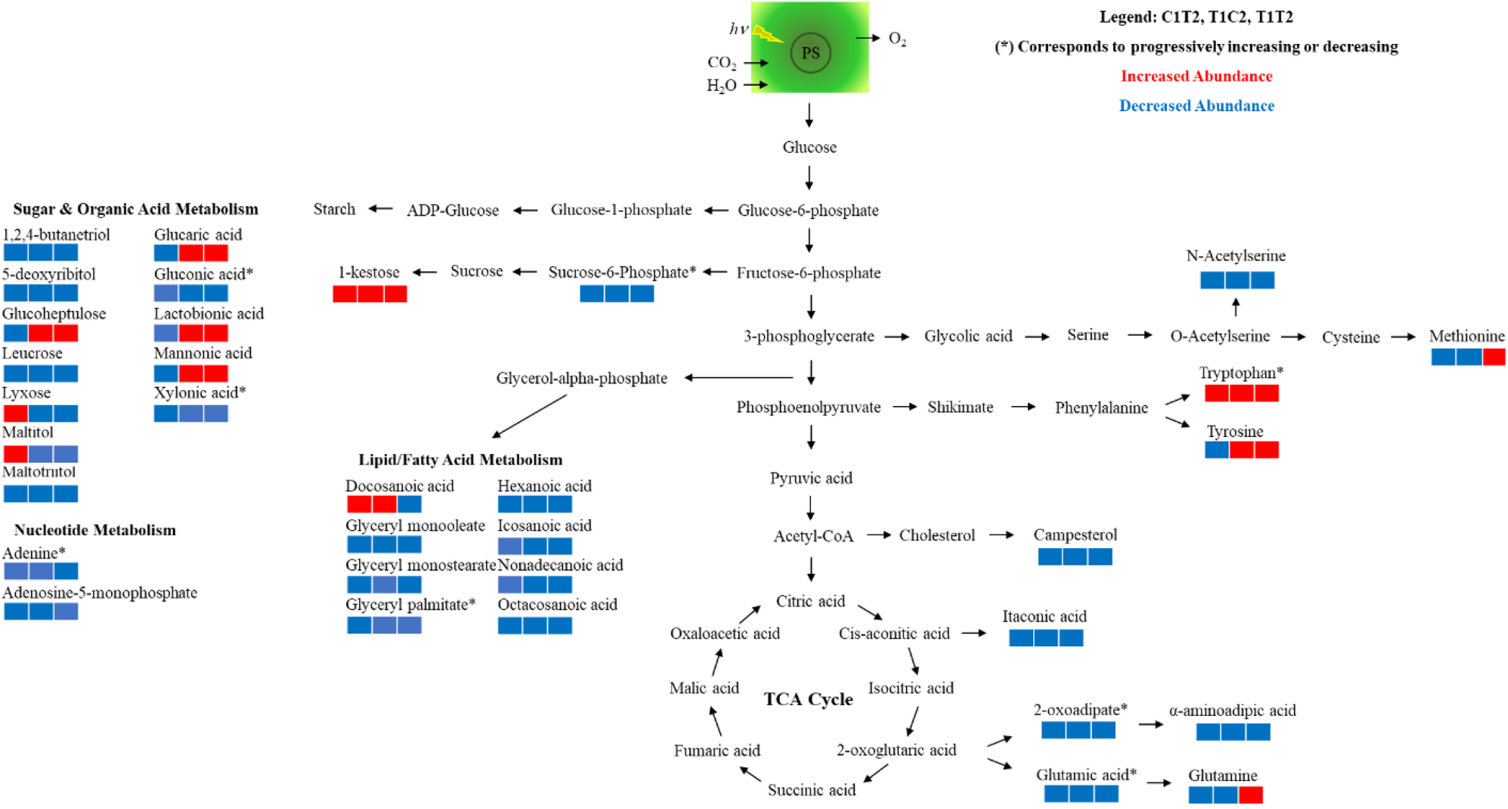
Metabolic pathway showing the changes in metabolite abundance (VIP score >1) in wheat grains harvested from plants exposed to perfluorooctanesulfonic acid (PFOS). Metabolites in red or blue font signifies increase or decrease, respectively, in C1T2-PFOS, T1C2-PFOS, and T1T2-PFOS compared to C1C2-PFOS. Analysis was performed using MetaboAnalyst 5.0. Refer to Figure 1 for the explanation of the treatments.

### Changes in Grain Metabolites

There were 12 metabolites (out of 34 total metabolites) that increased in abundance in previously treated grains; however, only tryptophan exhibited a progressive increase in abundances (e.g., 19%, 60%, and 107%), across all three generationally treated plants (C1T2-PFOS, T1C2-PFOS, and T1T2-PFOS, respectively) compared to the control (C1C2-PFOS) (Figure 6, Tables 3 and 4). Tryptophan is linked to the synthesis of secondary metabolites (e.g., indole-3-acetic acid) essential for growth and defense mechanisms and promoting grain yield in wheat.^31,49,50^ The metabolite 1-kestose (or 1-kestotriose) also showed a consistent increase in abundance (18–25%), albeit not a progressive increase, across all three generationally treated plants compared to the control. 1-kestose is the smallest inulin-type fructan necessary for the initiation of fructan synthesis in plants and according to recent studies, fructans function as endogenous, phloem-mobile stress signals during abiotic stress reactions.^51–53^ Past reports have found that fructans are one of the most significant carbohydrate reserves in vegetative tissues of wheat and are essential to the formation of wheat grains.^54^ The increase in tryptophan abundance in conjunction with the increase in 1-kestose abundance could be indicative of the plants’ adaptive mechanism for ensuring healthy grain yield. The other 10 metabolites (i.e., glucoheptulose, lyxose, maltitol, glucaric acid, lactobionic acid, mannonic acid, docosanoic acid, glutamine, tyrosine, and methionine) showed increased abundance only when the parent plants have been exposed to CeO_2_–NPs in one or two prior generations and PFOS in the third generation (Figure 6, Tables 3 and 4).

Interestingly, 22 out of 34 metabolites consistently decreased in abundance when plants were continuously exposed to CeO_2_–NPs and PFOS compared to control (Figure 6, Tables 3 and 4). These metabolites included important metabolites in grains such as lipids (campesterol, glyceryl monooleate, glyceryl monostearate, glyceryl palmitate, hexanoic acid, icosanoic acid, nonadecanoic acid, and octacosanoic acid), amino acids (2-oxoadipate acid, α-aminoadipic acid, glutamic acid, and N-acetylserine), and nucleic acids (adenine, adenosine-5-monophosphate). Notably, seven metabolites (i.e., sucrose-6-phosphate, gluconic acid, xylonic acid, glyceryl palmitate, 2-oxoadipate acid, glutamic acid, and adenine) exhibited progressive decrease in abundance when generationally exposed to CeO_2_–NPs and PFOS (i.e., C1T2-PFOS, T1C2-PFOS, and T1T2-PFOS) compared to the control (C1C2-PFOS) (Figure 6, Tables 3 and 4). These findings seem to be very significant because they provide evidence that continuous exposure of parent generations to CeO_2_–NPs could have more negative impacts on the metabolite compositions of the grains. A similar finding has been reported where a consistent generational exposure to CeO_2_–NPs resulted in progressive decrease in metabolite abundance of nicotianamide in wheat grains.^43^

The progressively decreasing abundances of seven metabolites (i.e., sucrose-6-phosphate, gluconic acid, xylonic acid, glyceryl palmitate, 2-oxoadipate acid, glutamic acid, and adenine) indicate that continuous exposure to CeO_2_–NPs of parent plants impacted storage reserves in wheat grains exposed to PFOS in the third generation. For example, glutamic acid is involved in the synthesis of arginine, proline, glutamine, γ-aminobutyric acid, and glutathione which play important roles in N metabolism and environmental stress tolerance in plants.^55–57^ Adenine is a key metabolite for energy and nitrogen metabolism and its continuous decrease across generations could signify metabolic and physiological changes in plants.^58,59^ The abundance for sucrose-6-phosphate, an intermediate metabolite for sucrose synthesis in plants,^60^ progressively decreased across treatments which could be an indication that sucrose synthesis or mobilization in plants was affected. Gluconic acid, xylonic acid, and glyceryl palmitate also decreased progressively across treatments which could indicate decreased resistance to stress when the plants have been continuously exposed to CeO_2_–NPs and PFOS. This is supported by the decreases, albeit not progressively, in seven (out of eight) lipid/fatty acid metabolites and other sugar and organic acid metabolites (Figure 6, Tables 3 and 4). These polyhydroxy (sugar and organic acid) and lipid/fatty acid metabolites are heavily involved in stress regulations in plants.^61,62^ The progressive decrease in such metabolites suggests that the generational exposure to CeO_2_–NPs, will reduce the nutritional quality of wheat, which may represent a health risk. Studies are needed to determine whether these NPs affect other Gramineae plants in the same way.

There are limited reports on the metabolomics of seeds or grains from plants exposed to PFOS, and there are even fewer reports in a generational exposure scenario with nanoparticles. As a result, there is no comparative discussion of acquired data regarding generational exposures to CeO_2_–NPs and PFOS effects on the grain metabolome. However, current results showed that abundances of sugar, nucleotide, organic acid, and amino acid metabolites in wheat grains were altered, but not the metabolites involved in the TCA pathway (Figure 6, Tables 3 and 4). In general, the abundances of most metabolites decreased (Figure 6, Tables 3 and 4), suggesting that continuous exposure to CeO_2_–NPs and PFOS could have negative implications for grain quality.

## Conclusions

This study shows that PFOS-exposed wheat grain’s metabolite composition, and therefore metabolic and biochemical processes, is significantly impacted by prior generations’ exposure to CeO_2_–NPs. Wheat subjected to CeO_2_–NPs for the first two generations followed by exposure to PFOS in the third generation has more pronounced alterations in the metabolic profile. However, the plant seems to recover from the prior generation’s treatments in the case of elemental uptake since T1T2 started to increase elemental accumulation again. Additionally, this study reveals that the growth and productivity of plants, especially during short exposure periods, may not be a strong predictor of plant’s responses to environmental contaminants. This was highlighted in our results on physiological and biochemical indices (i.e., chlorophyll content, lipid peroxidation, and enzyme activity), which showed little to no significant effects from the treatments. The data showed that PFOS was accumulated in the grains and that previous generation exposure to CeO_2_–NPs had no influence on its uptake and accumulation in wheat grains. However, grain metabolic and elemental profiles demonstrated subtle and invisible changes that may even impact the progeny generations. The findings support the idea that stress memory of plants can be transmitted to the next generation, highlighting the importance of understanding long-term impacts of parental exposure to contaminants of plants. Genomic studies, along with studies on epigenetic mechanisms and stress signaling pathways, are strongly recommended to understand the holistic interaction of NPs with plants through consecutive generations.

## Supplementary Material



## Notes

The elemental concentrations of roots and shoots (PDF)

## ⊥

Department of Environmental Health Sciences, UCLA Fielding School of Public Health, University of California Los Angeles, Los Angeles, CA 90095-1772;

## #

Department of Chemistry, University of Georgia, 140 Cedar Street, Athens, GA 30602;

## ∇

Department of Chemistry, University of Missouri, 601 S College Ave, Columbia, MO 65211;

## ○

Department of Chemistry, Missouri University of Science and Technology, 400 W 11th St., 223 Schrenk Hall, Rolla, MO 65409

## References

[1] EPA Technical Fact Sheet – Perfluorooctane Sulfonate (PFOS) and Perfluorooctanoic Acid (PFOA). Technical Fact Sheet - PFOS And PFOA; 2017, EPA 505–F-17–001.

[2] Zhang P., Ma Y., Zhang Z., He X., Zhang J., Guo Z., Tai R., Zhao Y., Chai Z. (2012). Biotransformation of ceria nanoparticles
in cucumber plants. ACS Nano.

[3] Hernandez-Viezcas J.
A., Castillo-Michel H., Andrews J. C., Cotte M., Rico C., Peralta-Videa J. R., Ge Y., Priester J. H., Holden P. A., Gardea-Torresdey J. L. (2013). In Situ
Synchrotron X-ray Fluorescence Mapping and
Speciation of CeO2 and ZnO Nanoparticles in Soil Cultivated Soybean
(Glycine max). ACS Nano.

[4] Rico C. M., Johnson M. G., Marcus M. A., Andersen C. P. (2017). Intergenerational
responses of wheat (*Triticum aestivum* L.) to cerium
oxide nanoparticles exposure. Environ. Sci.:
Nano.

[5] Zuverza-Mena N., Martínez-Fernández D., Du W., Hernandez-Viezcas J. A., Bonilla-Bird N., López-Moreno M.
L., Komárek M., Peralta-Videa J. R., Gardea-Torresdey J. L. (2017). Exposure of engineered nanomaterials
to plants: insights into the physiological and biochemical responses-A
review. Plant Physiol. Biochem..

[6] Wei L., Liu J., Jiang G. (2024). Nanoparticle-specific
transformations dictate nanoparticle
effects associated with plants and implications for nanotechnology
use in agriculture. Nat. Commun..

[7] Agathokleous E., Zhou B., Geng C., Xu J., Saitanis C. J., Feng Z., Tack F. M. G., Rinklebe J. (2022). Mechanisms
of cerium-induced
stress in plants: A meta-analysis. Sci. Total
Environ..

[8] Ghisi R., Vamerali T., Manzetti S. (2019). Accumulation
of perfluorinated alkyl
substances (PFAS) in agricultural plants: A review. Environ. Res..

[9] Gardea-Torresdey J. L., Rico C. M., White J. C. (2014). Trophic Transfer,
Transformation,
and Impact of Engineered Nanomaterials in Terrestrial Environments. Environ. Sci. Technol..

[10] Adu O., Ma X., Sharma V. K. (2023). Bioavailability,
phytotoxicity and plant uptake of
per-and polyfluoroalkyl substances (PFAS): A review. J. Hazard. Mater..

[11] Zhi Y., Lu H., Grieger K. D., Munoz G., Li W., Wang X., He Q., Qian S. (2022). Bioaccumulation and Translocation of 6: 2 Fluorotelomer
Sulfonate, GenX, and Perfluoroalkyl Acids by Urban Spontaneous Plants. ACS EST Eng..

[12] Stahl T., Heyn J., Thiele H., Huther J., Failing K., Georgii S., Brunn H. (2009). Carryover
of perfluorooctanoic acid
(PFOA) and perfluorooctane sulfonate (PFOS) from soil to plants. Arch. Environ. Contam. Toxicol..

[13] Rico C. M., Johnson M. G., Marcus M. A. (2018). Cerium
oxide nanoparticles transformation
at the root–soil interface of barley (Hordeum vulgare L.). Environ. Sci.:Nano..

[14] Costello M. C. S., Lee L. S. (2024). Sources, Fate, and
Plant Uptake in Agricultural Systems
of Per- and Polyfluoroalkyl Substances. Curr.
Pollut. Rep..

[15] Xu J., Cui Q., Ren H., Liu S., Liu Z., Sun X., Sun H., Shang J., Tan W. (2024). Differential uptake
and translocation
of perfluoroalkyl substances by vegetable roots and leaves: Insight
into critical influencing factors. Sci. Total
Environ..

[16] Zhang W., Tran N., Liang Y. (2022). Uptake of
per- and polyfluoroalkyl
substances (PFAS) by soybean across two generations. J. Hazard. Mater. Adv..

[17] Liu J., Wolfe K., Cobb G. P. (2019). Exposure to Copper Oxide Nanoparticles
and Arsenic Causes Intergenerational Effects on Rice (Oryza sativa
japonica Koshihikari) Seed Germination and Seedling Growth. Environ. Toxicol. Chem..

[18] Ogundele O. R., Fakunle M., Pope-Buss R., Churchman J., Akinwande B., Kirwa N., Ofoegbu P. C., Rico C. M. (2025). Physiological
and Metabolic Responses of Wheat (Triticum aestivum L.) after One-Generation
Exposure to Perfluorooctanesulfonic Acid (PFOS). ACS Agric. Sci. Technol..

[19] Shimalina N. S., Pozolotina V. N., Orekhova N. A. (2023). Stress memory in two generations
of Plantago major from radioactive and chemical contaminated areas
after the cessation of exposure. Int. J. Radiat.
Biol..

[20] Wang Q., Ebbs S. D., Chen Y., Ma X. (2013). Trans-generational
impact of cerium oxide nanoparticles on tomato plants. Metallomics.

[21] Tan W., Du W., Darrouzet-Nardi A. J., Hernandez-Viezcas J. A., Ye Y., Peralta-Videa J. R., Gardea-Torresdey J. L. (2018). Effects
of the exposure of TiO_2_ nanoparticles on basil (*Ocimum basilicum*) for two generations. Sci. Total Environ..

[22] Medina-Velo I. A., Zuverza-Mena N., Tamez C., Ye Y., Hernandez-Viezcas J. A., White J. C., Peralta-Videa J. R., Gardea-Torresdey J. L. (2018). Minimal
Transgenerational Effect of ZnO Nanomaterials on the Physiology and
Nutrient Profile of Phaseolus vulgaris. ACS
Sustainable Chem. Eng..

[23] Milenkovic I., Baruh Krstic M., Spasic S. Z., Radotic K. (2023). Trans-generational
effect of cerium oxide-nanoparticles (nCeO2) on *Chenopodium
rubrum* L. and *Sinapis alba* L. seeds. Funct. Plant Biol..

[24] Khan Z., Shahwar D., Khatoon B. (2022). Trans-generational
response of TiO2
nanoparticles in inducing variability and changes in biochemical pool
of lentil F2 progenies. J. Biosci..

[25] Rico C. M., Abolade O. M., Wagner D., Lottes B., Rodriguez J., Biagioni R., Andersen C. P. (2020). Wheat exposure to
cerium oxide nanoparticles
over three generations reveals transmissible changes in nutrition,
biochemical pools, and response to soil N. J.
Hazard. Mater..

[26] Xu Y., Du W., Yin Y., Sun Y., Ji R., He H., Yang S., Li S., Wu J., Guo H. (2022). CuO nanoparticles
modify bioaccumulation of perfluorooctanoic acid in radish (Raphanus
sativus L.). Environ. Pollut. Bioavailability.

[27] Wang X., Zhang W., Lamichhane S., Dou F., Ma X. (2023). Effects of
physicochemical properties and co-existing zinc agrochemicals on the
uptake and phytotoxicity of PFOA and GenX in lettuce. Environ. Sci. Pollut. Res..

[28] Xu Z., Tang T., Cheng H., Bao Q., Yu J., Zhang C., Wu T., Zhao X., Schramm K.-W., Wang Y. (2019). Negligible effects of TiO2 nanoparticles
at environmentally relevant
concentrations on the translocation and accumulation of perfluorooctanoic
acid and perfluorooctanesulfonate in hydroponically grown pumpkin
seedlings (Cucurbita maxima × C. moschata). Sci. Total Environ..

[29] Cai Y., Ma X., Yuan B., Fang G., Ullah H., Zhou D., Gao J. (2023). Metabolomics
of soybean (Glycine max L.) response to co-exposure
of pyrene and three metal oxide engineered nanomaterials. J. Hazard. Mater..

[30] Lichtenthaler H. K., Welburn A. R. (1983). Determination of Total Carotenoids and Chlorophylls
A and B of Leaf Extracts in Different Solvents. Biochem. Soc. Trans..

[31] Ofoegbu P. C., Wagner D. C., Abolade O., Clubb P., Dobbs Z., Sayers I., Zenobio J. E., Adeleye A. S., Rico C. M. (2022). Impacts
of perfluorooctanesulfonic acid on plant biometrics and grain metabolomics
of wheat (Triticum aestivum L.). J. Hazard.
Mater. Adv..

[32] Gay C., Gebicki J. M. (2000). A critical evaluation
of the effect of sorbitol on
the ferric-xylenol orange hydroperoxide assay. Anal. Biochem..

[33] Fiehn O., Wohlgemuth G., Scholz M., Kind T., Lee D. Y., Lu Y., Moon S., Nikolau B. (2008). Quality control for plant metabolomics:
reporting MSI-compliant studies. Plant J..

[34] Ma X., Wang Q., Rossi L., Ebbs S. D., White J. C. (2016). Multigenerational
exposure to cerium oxide nanoparticles: Physiological and biochemical
analysis reveals transmissible changes in rapid cycling Brassica rapa. NanoImpact.

[35] Blaine A. C., Rich C. D., Sedlacko E. M., Hundal L. S., Kumar K., Lau C., Mills M. A., Harris K. M., Higgins C. P. (2014). Perfluoroalkyl Acid
Distribution in Various Plant Compartments of Edible Crops Grown in
Biosolids-Amended soils. Environ. Sci. Technol..

[36] Felizeter S., McLachlan M. S., de Voogt P. (2012). Uptake of perfluorinated alkyl acids
by hydroponically grown lettuce (Lactuca sativa). Environ. Sci. Technol..

[37] Wen B., Li L., Liu Y., Zhang H., Hu X., Shan X.-Q., Zhang S. (2013). Mechanistic
studies of perfluorooctane sulfonate, perfluorooctanoic
acid uptake by maize (Zea mays L. cv. TY2). Plant Soil.

[38] Zhao H., Chen C., Zhang X., Chen J., Quan X. (2011). Phytotoxicity
of PFOS and PFOA to Brassica chinensis in different Chinese soils. Ecotoxicol. Environ. Saf..

[39] Zhao H., Guan Y., Zhang G., Zhang Z., Tan F., Quan X., Chen J. (2013). Uptake of
perfluorooctane sulfonate
(PFOS) by wheat (Triticum aestivum L.) plant. Chemosphere.

[40] Qian S., Lu H., Xiong T., Zhi Y., Munoz G., Zhang C., Li Z., Liu C., Li W., Wang X., He Q. (2023). Bioaccumulation
of Per- and Polyfluoroalkyl Substances (PFAS) in Ferns: Effect of
PFAS Molecular Structure and Plant Root Characteristics. Environ. Sci. Technol..

[41] Nason S. L., Thomas S., Stanley C., Silliboy R., Blumenthal M., Zhang W., Liang Y., Jones J. P., Zuverza-Mena N., White J. C., Haynes C. L., Vasiliou V., Timko M. P., Berger B. W. (2024). A comprehensive
trial on PFAS remediation: hemp phytoextraction
and PFAS degradation in harvested plants. Environ.
Sci.: Adv..

[42] Liu S., Zhou J., Guo J., Xue M., Shen L., Bai S., Liang X., Wang T., Zhu L. (2023). Impact Mechanisms of
Humic Acid on the Transmembrane Transport of Per- and Polyfluoroalkyl
Substances in Wheat at the Subcellular Level: The Important Role of
Slow-Type Anion Channels. Environ. Sci. Technol..

[43] Rico C. M., Wagner D., Abolade O., Lottes B., Coates K. (2020). Metabolomics
of wheat grains generationally-exposed to cerium oxide nanoparticles. Sci. Total Environ..

[44] Li P., Oyang X., Xie X., Guo Y., Li Z., Xi J., Zhu D., Ma X., Liu B., Li J., Xiao Z. (2020). Perfluorooctanoic acid
and perfluorooctane sulfonate co-exposure
induced changes of metabolites and defense pathways in lettuce leaves. Environ. Pollut..

[45] Rico C. M., Wagner D. C., Ofoegbu P. C., Kirwa N. J., Clubb P., Coates K., Zenobio J. E., Adeleye A. S. (2024). Toxicity assessment
of perfluorooctanesulfonic acid (PFOS) on a spontaneous plant, velvetleaf
(Abutilon theophrasti), via metabolomics. Sci.
Total Environ..

[46] Palmer C. M., Guerinot M. L. (2009). Facing the challenges of Cu, Fe and Zn homeostasis
in plants. Nat. Chem. Biol..

[47] Zhang Y., Du W., Zhang Y., Wang Z., Li H., Xia H., Fan S., Kong L. (2021). Metabolic analysis reveals temporal changes in C/N
metabolites and senescence of flag leaf in wheat during grain filling. Acta Physiol. Plant..

[48] Cui, Y. ; Wang, Z. ; Li, M. ; Li, X. ; Wang, S. ; Liu, C. ; Xin, D. ; Qi, Z. ; Chen, Q. ; Yang, M. ; Comparative Metabolomics Analysis of Seed Composition Accumulation in Soybean (Glycine max L.) Differing in Protein and Oil Content. Plant Cell Environ. 10.1111/pce.15448 40025870

[49] Radwanski E. R., Last R. L. (1995). Tryptophan biosynthesis
and metabolism: biochemical
and molecular genetics. Plant Cell.

[50] Raheem A., Shaposhnikov A., Belimov A. A., Dodd I. C., Ali B. (2018). Auxin production
by rhizobacteria was associated with improved yield of wheat (Triticum
aestivum L.) under drought stress. Arch. Agron.
Soil Sci..

[51] Livingston D. P., Hincha D. K., Heyer A. G. (2009). Fructan
and its
relationship to abiotic stress tolerance in plants. Cell. Mol. Life Sci..

[52] Van
den Ende W., De Coninck B., Van Laere A. (2004). Plant fructan
exohydrolases: a role in signaling and defense?. Trends Plant Sci..

[53] Vergauwen R., Van den Ende W., Van Laere A. (2000). The role of fructan in flowering
of Campanula rapunculoides. J. Exp. Bot..

[54] Schnyder H. (1993). The role of
carbohydrate storage and redistribution in the source-sink relations
of wheat and barley during grain filling — a review. New Phytol..

[55] Luo H., Duan M., Xing P., Zhang Y., Qi J., Kong L., Tang X. (2023). Effects of
L-glutamic acid application
on yield, grain quality, photosynthetic pigments, 2-acetyl-1-pyrroline,
and antioxidant system of aromatic rice. Field
Crops Res..

[56] Forde B. G., Lea P. J. (2007). Glutamate in plants:
metabolism, regulation, and signalling. J. Exp.
Bot..

[57] Hasanuzzaman M., Nahar K., Anee T. I., Fujita M. (2017). Glutathione in plants:
biosynthesis and physiological role in environmental stress tolerance. Physiol. Mol. Biol. Plants.

[58] Becana M. (2025). Recycling
of purine nucleotides in legumes: functional specialization of enzyme
isoforms in adenine salvage, cytokinin homeostasis, and nodulation
control. J. Exp. Bot..

[59] Haferkamp I., Fernie A. R., Neuhaus H. E. (2011). Adenine
nucleotide transport in plants:
much more than a mitochondrial issue. Trends
Plant Sci..

[60] Stein O., Granot D. (2019). An Overview of Sucrose Synthases
in Plants. Front. Plant Sci..

[61] Williamson J. D., Jennings D. B., Guo W.-W., Pharr D. M., Ehrenshaft M. (2002). Sugar Alcohols,
Salt Stress, and Fungal Resistance: Polyols—Multifunctional
Plant Protection?. J. Am. Soc. Hortic. Sci..

[62] He M., Ding N.-Z. (2020). Plant Unsaturated
Fatty Acids: Multiple Roles in Stress
Response. Front. Plant Sci..

