# Supplementary material for: Exposure of Wheat Plants to Cerium Oxide Nanoparticles for Two Generations Affects the Third Generation’s Responses to Perfluorooctanesulfonic Acid
Source: ACS Omega. 2025 Oct 1;10(40):47308–19. doi: 10.1021/acsomega.5c06292 (PMC12529381; doi:10.1021/acsomega.5c06292)
Supplement: Supplementary file 1 [file ao5c06292_si_001.pdf]

Supplemental Information for

Exposure of wheat plants to cerium oxide nanoparticles for two generations affects the third generation's responses to perfluorooctanesulfonic acid

Preston Clubb,[1] Riley Pope-Buss,[1] Maximo Reyes,[1] Jessica Linson,[1] Elim Horn,[2] Jose Peralta-Videa,[3] Illya Aidee Medina-Velo,[4] Cyren M. Rico[1]*

[1]Chemistry and Biochemistry Department, Missouri State University, 901 S National Ave, Springfield, Missouri 65897

[2]Willard High School, 515 E Jackson St, Willard, Missouri 65781

[3]Chemistry and Biochemistry Department, University of Texas at El Paso, 500 W University Ave, El Paso, Texas 79968

[4]Chemistry, Mathematics, and Physics Department, Houston Christian University, 7502 Fondren Rd, Houston, Texas 77074

*Corresponding author. Tel: 417 836 3304; Fax: 417 836 5507; E-mail address: CyrenRico@MissouriState.edu (C. M. Rico)

3 pages total with 2 tables

SI Table 1. Root elemental concentration (mg/kg) wheat generationally exposed to CeO_2-NPs and PFOS for 90 days. Values are mean ± SE ($n = 6$). Different letters across treatments indicate significant difference ($p < 0.05$). Refer to Figure 1 for the explanation of the treatments.

Elements	C1C2-PFOS	C1T2-PFOS	T1C2-PFOS	T1T2-PFOS
Mg	811 ± 71b	899 ± 67ab	752 ± 55b	1006 ± 69a
P	510 ± 55a	500 ± 22a	525 ± 63a	565 ± 10a
K	3817 ± 795a	4173 ± 1071a	4237 ± 842a	2729 ± 824a
S	1634 ± 110a	1562 ± 67a	1270 ± 111b	1369 ± 63ab
Ca	7172 ± 864ab	8707 ± 813a	5671 ± 853b	9385 ± 1034a
B	9.75 ± 0.66a	10.09 ± 0.99a	9.83 ± 0.63a	9.15 ± 1.03a
Cu	58 ± 17a	62 ± 6.3a	46.6 ± 16ab	12.3 ± 2.6b
Si	238 ± 29a	206 ± 8a	212 ± 11a	230 ± 7a
Mn	353 ± 25c	520 ± 59ab	403 ± 42bc	598 ± 82a
Zn	19 ± 2a	19 ± 1a	18.3 ± 1.8a	16.2 ± 0.8a
Fe	638 ± 37b	600 ± 57b	506 ± 64b	867 ± 57a
Co	1.96 ± 0.2c	2.7 ± 0.1ab	2.13 ± 0.2bc	3.1 ± 0.2a
Mo	0.42 ± 0.03a	0.42 ± 0.04a	0.43 ± 0.04a	0.47 ± 0.08a

SI Table 2. Shoot elemental concentration (mg/kg) wheat generationally exposed to CeO_2-NPs and PFOS for 90 days. Values are mean ± SE ($n = 6$). Different letters across treatments indicate significant difference ($p < 0.05$). Refer to Figure 1 for the explanation of the treatments.

Elements	C1C2-PFOS	C1T2-PFOS	T1C2-PFOS	T1T2-PFOS
Mg	2727 ± 183a	2791 ± 173a	2901 ± 180a	2860 ± 108a
P	202 ± 15a	180 ± 16a	184 ± 6a	207 ± 19a
K	28245 ± 1665a	30348 ± 1203a	28931 ± 1639a	28049 ± 1949a
S	3448 ± 396a	3850 ± 193a	3676 ± 307a	3896 ± 207a
Ca	6133 ± 493a	6220 ± 314a	6524 ± 271a	6561 ± 141a
B	8.71 ± 0.93b	8.16 ± 0.66b	10.15 ± 1.06ab	11.46 ± 0.39a
Cu	2.37 ± 0.16a	2.48 ± 0.16a	2.31 ± 0.13a	2.4 ± 0.15a
Si	163 ± 12b	182 ± 17ab	247 ± 31a	334 ± 101a
Mn	134 ± 14b	192 ± 13a	189 ± 22a	201 ± 4a
Zn	10.7 ± 1.7a	10.9 ± 1.4a	10.3 ± 0.7a	10.7 ± 1.9a
Fe	42 ± 4.5a	39 ± 1.6ab	33.4 ± 1.5b	33.4 ± 1.4b
Co	0.04 ± 0.01a	0.02 ± 0.01a	0.02 ± 0.01a	0.02 ± 0.01a
Mo	0.42 ± 0.16a	0.16 ± 0b	0.2 ± 0.02ab	0.13 ± 0.01b
